# Harm reduction in practice – The Community Oriented Substance Use Programme in Tshwane

**DOI:** 10.4102/phcfm.v12i1.2285

**Published:** 2020-05-06

**Authors:** Andrew Scheibe, Shaun Shelly, Johannes Hugo, Matilda Mohale, Sasha Lalla, Wayne Renkin, Natasha Gloeck, Senzo Khambule, Lorinda Kroucamp, Urvisha Bhoora, Tessa S. Marcus

**Affiliations:** 1 COPC Research Unit, Department of Family Medicine, School of Medicine, Faculty of Health Sciences, University of Pretoria, Tshwane, South Africa; 2 Tshwane Leadership Foundation, University of Pretoria, Tshwane, South Africa

**Keywords:** Harm reduction, Substance use, Community oriented primary care, Behavioural health management, Family medicine, Primary healthcare, Public health

## Abstract

**Background:**

The Community Oriented Substance Use Programme (COSUP) is the first publicly funded, community-based programmatic response to the use of illegal substances in South Africa. It is founded on a systems thinking, public health and clinical care harm reduction approach.

**Aim:**

To describe the critical components, key issues and accomplishments in the initiation and delivery of evidence-based, community-oriented, substance-use health and care services.

**Setting:**

The Community Oriented Substance Use Programme is implemented by the University of Pretoria in four of seven Tshwane Metropolitan Municipality regions.

**Methods:**

Quantitative and qualitative data were extracted and triangulated from plans, reports, minutes and other documents.

**Results:**

Between 2016 and 2019, COSUP engaged in national and local policy and guidelines development. In Tshwane, it created practical working relations with 169 organisations and institutions and set up 17 service sites. These provide counselling, linkage to care and opioid substitution therapy services to 1513 adults (median age of 30 years), most of whom are male (90%), with similar proportions of clients who smoke (51%) or inject (49%) heroin. It also offers needle and syringe services (approximately 17 000 needles distributed/month) and has built human resource capacity in harm reduction among staff, clients and personnel in partner organisations.

**Conclusion:**

The Community Oriented Substance Use Programme offers an evidence-based, public-health informed, feasible alternative to an abstinence-based approach to substance use. However, to translate the programme’s achievements into sustainable outcomes at scale requires health system integration; generalist, patient-centred care; affordable medication in a comprehensive package of harm reduction services; multisectoral partnerships; systematic, continuous capacity development; financial investment; and sustained political commitment.

## Introduction

South Africa (SA) faces many intersecting health and social challenges, among which is the perception of the widespread presence and broad-ranging adverse effects of substance use, specifically heroin.^1^ Although there is little published data on the prevalence of the use of heroin in Tshwane, known locally as *nyaope*,^i^ recent reports show increasing heroin trafficking, heroin availability and heroin-related drug treatment admissions.^1,2^ The injecting of heroin, including needle reuse and sharing, has been noted.^3^ A recent study identified high levels of human immunodeficiency virus (HIV), hepatitis C virus (HCV) and HIV-HVC co-infection among people who inject drugs in Tshwane (38%, 67% and 27%, respectively, *n* = 324).^4^

In 2016, the Department of Family Medicine (DFM, University of Pretoria) was requested by the City of Tshwane to develop the Community Oriented Substance Use Programme (COSUP). It is the first publicly funded, community-based programmatic response to the use of unregulated drugs in SA. In the last half of 2015, cannabis, heroin, cocaine and methcathinone were the most common primary substances of use among patients registered at substance use treatment centres in Gauteng (38%, 5%, 4% and 4%, respectively).^2^,^ii^

In a significant break with prohibitionist and abstinence-focused assumptions and practices that have yielded poor outcomes,^5^ COSUP is founded on a systems thinking, harm reduction approach to public health and clinical care.^6^ It seeks to provide a continuum of evidence-based, substance-use services that are integrated into the delivery of community-oriented primary care (COPC).^7^ The core service package includes physical, mental and substance use screenings, assessments, brief interventions and referrals; harm reduction counselling; opioid substitution therapy (OST)^iii^; and needle and syringe services as well as social services, skills development and shelter.^6^ The HIV and tuberculosis (TB) screening has been integrated into the package, and viral hepatitis testing is conducted where laboratory services and resources allow. Treatment of HIV, TB and HCV infections is provided in partnership with the available health services.

Like all healthcare interventions that involve novel approaches and new practices and procedures, COSUP engages actively in capacity development. Responses to unregulated drug use are shrouded in prejudice and fear of contagion, as well as ignorance and uncertainty, much like TB in the 19th century and HIV in the twentieth century.^8^ Because of this, the programme also has developed and proactively applied a number of processes and organisational measures to counter the moral and political opposition to harm reduction programmes and to engage policy reform.^9^

## Community Oriented Substance Use Programme implementation – The practice

The Community Oriented Substance Use Programme was initiated in mid-2016 with the signing of a 3-year service level agreement between the City of Tshwane and the University of Pretoria. Understood as a test of concept, the programme has adopted a staggered start, adaptive planning approach^10^ to designing and implementing the different elements of the programme.

## Mapping the organisational platform

Implementation began with mapping the people and organisations involved in substance use management in Tshwane. Over the 3 years, through workshops, meetings and community forums across the city, COSUP was able to identify potential partners and develop practical working relations with workers and administrators from 169 health, law enforcement, social service, housing, social justice and other organisations and institutions. Essential to the effectiveness of the intervention, this work is ongoing and takes time and effort, not least of all because many participants come schooled in the ‘common sense’ of abstinence and are strongly opposed to harm reduction as an approach to substance use management.

## Sites

Community Oriented Substance Use Programme service sites have been phased in over the 3 years. Seven were set up and running by June 2017. By mid-2019 there were 17 functional and viable sites available to clients in Tshwane. Twelve included drop-in centres that provided access to ablution facilities, nutrition, computers, other psychosocial services and/or safe spaces to socialise.

The initial idea of setting up sites in public health service facilities proved challenging to realise in many instances. Much like in the early years of HIV treatment provision, space constraints, bureaucracy, stigma and public safety concerns forced COSUP to establish most sites outside the public primary healthcare facilities. The majority (*n* = 10) operate within or from the premises of non-profit and faith-based organisations. Five sites are in public health facilities. Of these, three are in primary healthcare clinics, one of which is jointly run by the University of Pretoria and two are on hospital premises, where they assist with inpatient care of people with substance use problems in the presence of co-morbidities. The remaining two sites operate in private healthcare practices.

Identifying and securing sites had a direct impact on the start dates, costs of setting up and the overall number of service sites. It added to the challenges of managing multi-morbidity in the substance-using population because additional linkage to care processes and practices had to be created. Also, it detracted from the programme’s efforts to normalise the provision of harm reduction services in the public healthcare system. On the positive side, it created a multisectoral foundation for substance use services beyond the domain of rehabilitation.

## Staff

**FIGURE 1:**
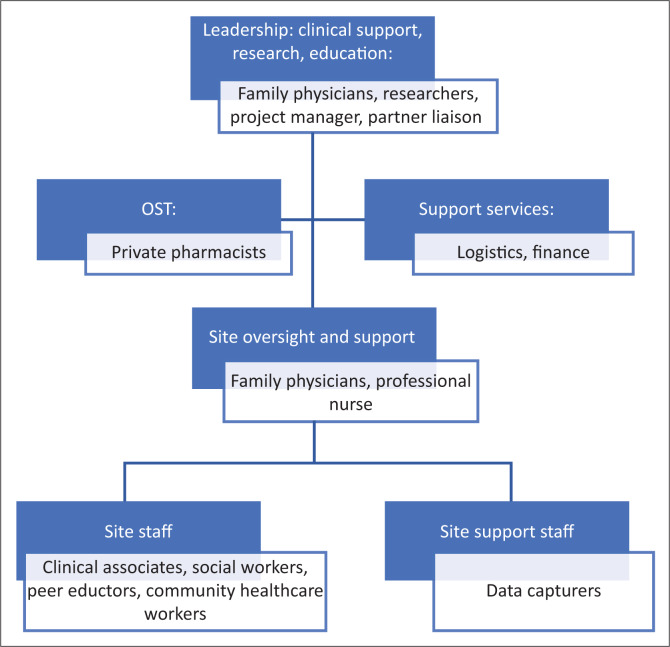
Staffing structures for Community Oriented Substance Use Programme.

The Community Oriented Substance Use Programme project and service teams were created parallel to and during the process of site identification and set-up. The project team ‘Backoffice’ comprises project managers, partner liaison and support staff (*n* = 9), staff supervisors (*n* = 2) and researchers (*n* = 3 part-time). Between three and five family physicians and a professional nurse provide clinical oversight and support across the sites. At each site, there is a service team of up to seven staff members, comprising a social worker, a mid-level medical practitioner, clinical associate and one or more peer educators who are recruited from the community of people who use drugs. Some teams also include one or more community health workers (CHWs) who work in the government district health system. Data capturers (*n* = 10) support several sites. A schematic representation of the staffing is included in Figure 1.

Over the 3 years, the programme has trained and employed 121 personnel, with 91 being in employment at the end of the first quarter of 2019. Although attrition is standard in programmes like this, a critical preventable contributor to the loss of trained personnel relates to conditions of employment, including the challenges of employing people who actively use drugs, the insecurity of annual contracts and uncertainties around funder commitment beyond the 3 years.

## Services

In a harm reduction intervention, delivering services means operating simultaneously at many levels and across different sectors.

The most immediate, direct deliverable is the provision of professional health and care services to people who use substances. To ensure quality care, COSUP has developed standard operational procedures for all levels of staff for its services, from client selection criteria for intake to voluntary or involuntary termination and effective OST.

Integrated medical, psychological and social screening is part of condition identification and initial care as an optional ongoing component of the services offered to clients. The programme uses several health-screening tools. For example, CHWs screen households and individuals for substance use in their homes by using a custom-designed application to support community-based service delivery (AitaHealth™). To screen for the presence and assess the risks of substance use and to provide linked brief interventions,^iv^ team members at all levels use the Alcohol, Smoking and Substance Involvement Screening Test (ASSIST 3.0) developed by the World Health Organization (WHO). Because of service lag time, the need to integrate data across systems and services, and the practical inefficiencies of using a paper version, COSUP worked with the University of Adelaide and Opentute™ to adapt and operationalise an electronic version of ASSIST (eASSIST) to enable integration with AitaHealth™ and COSUP’s patient management system (Synaxon™).

From the start of service delivery, screening results have been used to select the services offered clients, including brief interventions, risk reduction strategies, support for better practices, self-regulation, OST, individual psychotherapy, harm reduction counselling, practical support, psychosocial group therapy and referral for acute or chronic medical treatment. Testing services for HIV infections were included from the second year of the programme, and people diagnosed with HIV infection were referred to other providers for treatment. The patient management system was initially designed to focus on OST and efforts are ongoing to better capture information around HIV and TB testing and the HIV and TB treatment cascades.

The programme helped create 19 community advisory groups to ensure person-centredness, equity and inclusivity. Community advisory groups provide a voluntary forum for staff, clients and other stakeholders to meet to identify and respond to issues and problems that arise from the programme, or the context in which it operates. The purpose and frequency of meetings vary across the sites, as their functionality depends on participant interest and needs.

The Community Oriented Substance Use Programme has also responded to endemic social issues arising from homelessness, the need for livelihoods and family conflict and disruption. These all require society-wide interventions and are critical to harm reduction outcomes. Although the programme works with the Tshwane Leadership Foundation, Sediba Hope, the University of Pretoria’s Community Engagement and other partner organisations to secure transitional shelter and develop the skills and work readiness of clients, satisfactorily addressing these issues is beyond the immediate scope of the current programme.

## Opioid substitution therapy and needle and syringe services

Accessible OST and needle and syringe services are fundamental to the success of the sites, service delivery and effective patient care.^8^ Although well understood by the programme, there has been a failure to allocate funding for their provision, and system constraints have meant that neither methadone nor buprenorphine, the medications used to treat heroin dependence, are on SA’s Essential Medicines List (EML) for maintenance (largely because of political reasons).^9,11^

The process to obtain methadone on the EML is at an advanced stage and COSUP has been used as the practical example for both the need and the possibility to implement OST at scale.

In terms of OST, COSUP was granted permission to procure methadone from the private sector in May 2017 and 120 clients were initiated on OST in June. At the cost of approximately R2400 (US$160) per month per person for the recommended dose, COSUP could provide only free OST to a few clients (*n* = 34) through approved reallocation of a limited amount of programme funds. It was also able to provide OST to another 86 who could afford to pay out of pocket. As seen in other South African settings,^11^ during that year, the unaffordability of methadone led to threefold lower retention among clients who self-funded their treatment compared with those whose treatment was funded by COSUP. There was a general drop-off in engagement with the other services offered to heroin-dependent people at sites. And like elsewhere,^12^ the out-of-pocket payment cost led to sub-optimal dosing as well as a reduction in the duration and protective benefits against overdosing among those who are able to pay. At the end of the programme’s third year, 5861 people had used a COSUP service at least once. A total of 1513 (26%), mostly male (*n* = 1366; 90%), services users were started on OST. With a median age of 30 years (interquartile range: 26–34), 744 (49.2%) injected and 769 (51.8%) smoked heroin. More than half (57%) of OST clients were retained for at least 6 months. Although this rate is similar to OST retention rates in other low- and middle-income settings, it continues to be adversely influenced by the fact that OST is neither free nor affordable.^10^ Other factors contributing to loss to follow-up were suboptimal methadone dose, residing outside of the city centre and injecting heroin.

The programme has had to grapple with a number of practical service delivery issues, including establishing procedures for the safe storage of dispensed methadone at the sites and finding ways for stable clients to take doses of methadone at their homes.

Through interactions with international experts at SA Drug Policy Week 2018,^13^ the programme reviewed OST dosing, down titration practices and initial assumptions of time-limited OST. Staff adjusted dosing to meet international standards after realising the negative effects of under-dosing and premature down titration. They also began to engage system reluctance to encourage long-term OST intervention as well as mistaken beliefs among clients and their families about the ‘addictive’ nature of and high-dose risks of methadone. Community Oriented Substance Use Programme, together with the South African HIV Clinicians Society, the WHO, TB HIV Care, Durban University of Technology and the University of Cape Town, is advocating for the inclusion of methadone and buprenorphine on the South African EML for use at all levels of care. Once included and made available in the public sector, increased demand can be expected to reduce price. Furthermore, to increase competition and lower costs, this group has encouraged the registration of additional OST medications into the South African market. It is also engaged in the development of National Department of Health OST Clinical Guidelines and a National OST Implementation Plan. In 2019, COSUP found itself unable to sustain existing services, provide adequate dosing or initiate new clients and thereby meet the expanding demand for OST with serious negative ethical and practical implications for the programme and the public healthcare in general.

The diversion of methadone is often given as a reason for restricting access. However, the diversion of methadone at the patient level is often the result of inadequate coverage^14^ or is motivated by altruism,^15^ and diverted methadone can assist people to make the decision to enter programmes.^16^ With the provision of OST through private pharmacies to the sites and clients, there is no diversion in the supply chain.

## Needle and syringe services

Research shows that the provision of sterile needles to people who inject drugs reduces blood-borne infections like HIV and HCV, is a critical intervention to support community concerns around hazardous waste pollution and is especially useful when needle and syringe services form part of a harm reduction package provided at multiple levels and in multi-component programmes.^4^ Since March 2018, after piloting in one site, needle and syringe services have been running from all COSUP sites. Uptake by people who inject drugs is evidenced by an average monthly distribution of sterile needles that has grown from an initial 6000 to more than 17 000, with more than 85% of used needles returned. Community Oriented Substance Use Programme adds experience that needle and syringe services are highly acceptable among people who inject drugs. Within COSUP, the needle and syringe service is constrained by ineffective procurement of injecting equipment.

## Intersectoral cooperation

It is also vital to ensure that possession of injecting equipment, including sterile water, is not used by law enforcement to arrest people who inject drugs or COSUP service providers. To improve trust between service providers, people who use drugs and the community, COSUP has facilitated significant intersectoral cooperation. It has created a multi-disciplinary team of local representatives from the South African Police Service (SAPS), Lawyers for Human Rights, Tshwane Leadership Foundation, Tshwane District Hospital and the University of Pretoria’s Community Engagement, to address common concerns for safety, security and well-being. The programme has also supported capacity development, especially within law enforcement. Community Oriented Substance Use Programme staff members have proactively sensitised police around the need for harm reduction and developed the police’s understanding of this approach through regular meetings and workshops as well as specific interventions. For example, ‘sports is our gang’ soccer and netball-friendly tournaments provided the opportunity for mixed teams of players from local police stations and COSUP clients to participate in a 2-day competition. At a critical, if more mundane service level, SAPS has started to call on COSUP staff to assist with getting health and care services to people in their custody. Community Oriented Substance Use Programme and its partners engage with SAPS when their programme staff or clients are wrongfully treated, threatened or attacked. Amongst other things, COSUP integrates stigma mitigation^13^ into internal and external meetings and processes and has provided additional sensitisation training to reduce the well-known exclusory effects of prejudice and discrimination in access to and utilisation of health and others services. Despite all these efforts, instances of police confiscating injecting material still take place.

In 2019, to assist the City of Tshwane to fulfil its municipal mandate, COSUP supported the re-establishment of regional drug action committees by helping with planning, monitoring and evaluation of the committees through the development of standard operating procedures and training.

## Training, education and research

The Community Oriented Substance Use Programme is developing #Learning@COSUP. It uses a capability approach through which learning becomes an ongoing process involving clients, families, staff and leadership. The programme began by training doctors, clinical associates, social workers and peers newly recruited into the team. It mobilised available expertise in harm reduction, substance use disorder management, community-orientated primary care and learning. Individual and team competencies were developed and grown over time through work-integrated learning that involved practice, weekly team meetings to review issues in service delivery and capacity development around the meaning of non-stigmatising service delivery and client agency, as well as OST and needle and syringe service provision.

Also, the programme provided in situ training for social development, police and healthcare professionals and workers, including doctors, nurses, allied health professionals and CHWs. In the hospital setting this has meant guiding doctors on OST prescribing, training ward staff on harm reduction, the importance of OST provision and adherence, stigmatisation and linkage to care.

Apart from the skills provided to clients to manage their substance use more safely, COSUP enabled 800 clients to participate in life skills, work readiness and other skills development programmes offered by partners. About half of those trained were able to find some form of formal, albeit temporary, employment.

Through COSUP it has also been possible to support the integration of harm reduction principles and evidence-based substance use treatment services into medical and social worker professional education. At the University of Pretoria undergraduate level, harm reduction and exposure to substance use services are now included in medical student rotations in first, fourth and fifth years as well as in the orientation, ethics and research methods courses of clinical associate and social work students. Identifying, understanding and responding to harmful substance use are also included in postgraduate medical registrar training and are a focus of research for several honour’s, master’s and doctoral students in social work and family medicine.

Overall, COSUP has grown the competency base in substance use service delivery by using a harm reduction approach. With more than 3000 student contacts, this intervention has put SA at the forefront of substance use curriculum reform in the region. It has also stimulated growing academic and practical interest in researching and responding to harmful substance use across the academic complex.

A summary of COSUP outputs and factors influencing the project is included in Box 1.

BOX 1 Summary of Community Oriented Substance Use Programme outputs and factors influencing implementation.

**Project outputs (2016–2019):**

- Programme implemented in four regions of Tshwane
- 17 sites established, and fostered relationships with 169 organisations, employing 121 people and engaging more than 3000 students
- 19 community advisory groups established
- 5861 people accessed COSUP services and 1513 people initiated onto OST
- ±17 000 needles and syringes distributed monthly
- Progress towards integration of a mobile phone application, electronic patient management system and an electronic ASSIST platform
- Informed national OST guidelines and implementation plan

**Table:** 

Enabling factors	Challenges
Paradigm shift to harm reduction	Healthcare worker attitudes
Intersectoral collaboration	Law enforcement attitudes
Availability of OST medication	Inflated cost of OST medication
Accessible comprehensive treatment services at community level	Sustainability of funding
Focus on training	Limited capacity and experience in evidence-based treatment of opioid use disorders and harm reduction
Empowerment of communities who use substances	Community member attitudes
Advocacy for system change and reduction of medication cost	-

COSUP, Community Oriented Substance Use Programme; OST, opioid substitution therapy.

## Conclusion

The Community Oriented Substance Use Programme is a harm reduction, community-oriented, primary healthcare, systems approach to substance use. In its short existence, the programme has shown that it is feasible to respond to harmful substance use in a way that improves the health and well-being of people who use drugs, encourages service cooperation around substance use and improves role players’ understanding of the systemic issues that make managing and responding to substance use so complex without the singular focus on abstinence.

Implementing COSUP has helped distil critical issues that need addressing to make it sustainable and scalable. Key among these include ensuring:

1. core healthcare service deliverables – system integration and generalist, patient-centred care
2. affordable medication in a comprehensive package of harm reduction services that includes OST and needle and syringe services
3. multiple partner involvement across services and sectors to meet the many-sided needs of patients, clients, families and communities and to find and share ways of responding cooperatively to inevitable systemic disorder and unpredictability
4. systematic, continuous human resource capacity development in harm reduction and community oriented health and care service delivery
5. financial investment in a harm reduction programmatic response to substance use
6. sustained political commitment so that the investment is realised in positive health and social outcomes
7. clear and significant change from the war on drugs towards a human rights approach including diversion programmes from the law-enforcing sector.

## Footnotes

i Other terms for heroin in different parts of South Africa include *whoonga, wunga, unga*, sugars and pinch.

ii Surveillance of substance use is limited to the South African Community Epidemiology Network on Substance Use and reflects data from people who access substance use treatment centres linked to the network. Data are reported on a provincial level.

iii Opioid substitution therapy as a maintenance therapy is the gold standard treatment for opioid use disorder. It involves the long-term provision of an opioid agonist medication by a clinician at the correct dose in a structured environment, with access to voluntary psychosocial services. Opioid substitution therapy reduces mortality (notably overdose) and morbidity (especially a reduction in HIV and viral hepatitis infections through reduced injecting practices) and improves well-being and social functioning.

iv The quantification of moderate- and high-risk substance use, based on ASSIST scores, has yet to be done.
